# Supplementary material for: Disparities in behavioral health and experience of violence between cisgender and transgender Thai adolescents
Source: PLoS One. 2021 May 28;16(5):e0252520. doi: 10.1371/journal.pone.0252520 (PMC8162664; doi:10.1371/journal.pone.0252520)
Supplement: Attachment — Submitted filename: Response to Reviewers.docx [file pone.0252520.s004.docx]

Response to Reviewers

PONE-D-20-21445

Disparities in Behavioral Health, Experience of Violence, and Problematic Parental Behaviors Between Cisgender and Transgender Thai Adolescents

| **Comment** | **Response** |
| --- | --- |
| **Journal Requirements:** |  |
| 1. Please ensure that your manuscript meets PLOS ONE's style requirements, including those for file naming. The PLOS ONE style templates can be found at  https://journals.plos.org/plosone/s/file?id= wjVg/PLOSOne_formatting_sample _main_body.pdf and  https://journals.plos.org/plosone/s/file?id= ba62/PLOSOne_formatting_sample_title _authors_affiliations.pdf | Changes made |
| 2. Our internal editors feel that your manuscript is within the scope of our Health and Health Care in Gender Diverse Communities Call for Papers. The final collection of papers will be selected by a team of Guest Editors for PLOS ONE: Asa Radix (New York University/Langone Health), Jae Sevelius (University of California San Francisco), and Ayden Scheim (Drexel University). Additional information can be found on our announcement page: https://collections.plos.org/s/health-gender-diversity. If you would like your manuscript to be considered for this collection, please let us know in your cover letter and we will ensure that your paper is treated as if you were responding to this call. If you would prefer to remove your manuscript from collection consideration, please specify this in the cover letter. | We agree with the suggestion of internal editors and have indicated our wish for the manuscript to be considered for the Health and Health Care in Gender Diverse Communities collection in the cover letter accordingly |
| 3.We note that you have indicated that data from this study are available upon request. PLOS only allows data to be available upon request if there are legal or ethical restrictions on sharing data publicly. For information on unacceptable data access restrictions, please see http://journals.plos.org/plosone/s/data-availability#loc-unacceptable-data-access-restrictions.    In your revised cover letter, please address the following prompts:    a) If there are ethical or legal restrictions on sharing a de-identified data set, please explain them in detail (e.g., data contain potentially identifying or sensitive patient information) and who has imposed them (e.g., an ethics committee). Please also provide contact information for a data access committee, ethics committee, or other institutional body to which data requests may be sent.    b) If there are no restrictions, please upload the minimal anonymized data set necessary to replicate your study findings as either Supporting Information files or to a stable, public repository and provide us with the relevant URLs, DOIs, or accession numbers. Please see http://www.bmj.com/content/340/bmj.c181.long for guidelines on how to de-identify and prepare clinical data for publication. For a list of acceptable repositories, please see http://journals.plos.org/plosone/s/data-availability#loc-recommended-repositories.    We will update your Data Availability statement on your behalf to reflect the information you provide. | After internal discussions, we are pleased to inform the editors that the data from this study are no longer subject to restrictions. We will upload the anonymized data set necessary to replicate the study findings as a Supporting Information file. Please kindly update our Data Availability statement. |
| 4. Please include captions for your Supporting Information files at the end of your manuscript, and update any in-text citations to match accordingly. Please see our Supporting Information guidelines for more information: http://journals.plos.org/plosone/s/supporting-information. | The Supporting Information files will be updated accordingly. The caption reads as follow:  “**S1 File. Anonymized Data Set.** Anonymized data set to replicate the study findings”. |
| **Reviewers' comments:** |  |
| **Reviewer #1:** |  |
| This paper sought to deal with an important topic, the disparities in health and behavioral outcomes between cisgender and transgender adolescents in Thailand. However, this paper has several critical weaknesses. | We would like to thank the reviewer for the very useful comments and insights. We have made our best attempt to revise the manuscript according to the reviewer’s suggestions |
| ***** Major comments** |  |
| Comment 1.  A notable strength of this study is that it compares a wide variety of health outcomes, health-related behaviors, and experiences between cisgender and transgender Thai adolescents using a large, nationwide sample of school-going Thai adolescents. The study’s outcomes could be categorized into three groups.  Group 1: mental health and health behaviors  Group 2: experiences of violence  Group 3: parental addictive and violent behaviors  Currently, the results for the three groups are presented together in table 3. Analyses regarding these variables should be revisited to take into consideration for their differing implications.  First of all, it is strongly advised that the author drops variables in group 3 – parental addictive and violent behaviors – for a couple of reasons. Group 3 variables are different from group 1 and 2 variables in that they are not about the participants themselves, but about their parents. More importantly, the conclusions drawn on parental behaviors by the author (lines 400-407, page 31) can potentially be interpreted to blame transgender youths for their parents’ addictive and violent behaviors. For these reasons, the reviewer strongly recommends the author to drop group 3 variables from the study. This could be a research topic for another paper.  Second, the reviewer recommends that the results for groups 1 and 2 are presented in separate tables. Variables in group 1 are indicators of participants’ health, and variables in group 2 are about experiences of violence. It is highly possible that the experience of group 2 could be used to explain the observed health disparities of the group 1 results. It is suggested that the author first presents the results from group 1 variables and describes the health disparities between cisgender and transgender Thai adolescents. This should then be followed by results from group 2 variables in a separate table, with the discussion of these results in the context of health disparities shown in group 1 results. | We wish to thank the reviewer for the detailed and constructive comments.  We agree with the reviewer’s comment regarding parental addictive and violent behaviors and have dropped the variables from Table 3. We have also deleted the paragraph regarding parental behaviors in lines 400-407, page 31.  We have also separated Table 3 into the new Table 3 (for Group 1 variables: mental health outcomes and health behaviors) and Table 4 (for Group 2 variables: experiences of violence). We have also added discussions regarding results of Table 4 (Group 2 variables) in the context of Group 1 variables into the DISCUSSION section as follows:  “A study on Thai transgender women's experiences of stigma in daily lives showed various experiences of verbal, psychological and physical violence[11]. Although the Thai education has introduced sexuality education in its curriculum, such effort may not be effective in reducing homonegative attitudes among students[12]. These higher exposure to violence among transgender compared to cisgender respondents could be among the influences of the disparities in health behaviors.” |
| Comment 2.  Although the results clearly show the health disparities experienced by transgender youths in Thailand, this paper did not provide sufficient information about the hostile social environment against transgender youth in Thailand. The author does briefly mention the negative social situations transgender youths in Thai face in the introduction (lines 57-67, pages 4-5). It is suggested that the author includes specific examples of unfair social and institutional conditions Thai transgender youths experience to better contextualize the main results. | We have revised the INTRODUCTION section. Another reviewer suggested the inclusion of the following references, which we have followed and mentioned examples of social and institution conditions that Thai transgender youths experiences:  de Lind van Wijngaarden JW, Fongkaew K. “Being Born like This, I Have No Right to Make Anybody Listen to Me”: Understanding Different Forms of Stigma among Thai Transgender Women Living with HIV in Thailand. Journal of Homosexuality. 2020 Aug 26:1-8.  Manalastas EJ, Ojanen TT, Torre BA, Ratanashevorn R, Hong BC, Kumaresan V, Veeramuthu V. Homonegativity in southeast Asia: Attitudes toward lesbians and gay men in Indonesia, Malaysia, the Philippines, Singapore, Thailand, and Vietnam. Asia-Pacific Social Science Review. 2017 Jun 1;17(1):25-33. |
| Comment 3.  In the paper, assigned female at birth (AFAB) and assigned male at birth (AMAB) are categories of participants who did not provide an answer for the question on their gender identity. According to the paragraph that starts in line 353 of page 29, author also mentioned that they might be the people who did not understand why a separate question was asked on gender or the people who do not want reveal their gender identity, and people with missing responses. So it is evident that authors do not have enough information to understand who AFAB and AMAB actually are. Therefore, it is impossible to discuss about their findings. It is strongly recommended that the author excludes those with missing gender information from the analysis. | We agree with the reviewer and have excluded those with missing gender information from the analyses, and deleted the AFAB and AMAB columns from Table 3 and Table 4 accordingly. We have also revised the corresponding parts in the METHODS section and retained the following remarks at the end of the second paragraph of DISCUSSION:  “However, approximately one-sixth of the respondents did not answer the gender identity question. The Thai word in the questionnaire was ‘*phet withi*’, which was not part of vernacular Thai. It is possible that respondents who did not answer the gender identity question either did not understand it or perceived it to be a duplicate of the question pertaining to sex assigned at birth and decided to skip it. Future studies should consider changing the sex assigned at birth question may be changed from "Sex..." to "What sex were you assigned at birth?". Future studies should consider modification of the gender identity measurement question to help reduce this non-response, e.g., changing from "You think you are..." to "What gender do you identify as?" to reflect the notion that gender identity is firmly felt and integral to one's being.” |
| Comment 4.  This paper compares health statuses of Thai transgender adolescents only against their cisgender male counterparts (reference group). There is no rationale as to why cisgender male adolescents were chosen as the sole reference group to investigate health statuses of adolescents of different gender identities. Please provide a rationale as to why the author chose cisgender male adolescents as the reference group. In the events that the author cannot provide the rationale, it is suggested that they review previous works that have compared cisgender and transgender health outcomes (Downing & Przedworski, 2018; Lee et al., 2020; Thoma et al., 2019) and revise the analyses accordingly.  Downing, J. M., & Przedworski, J. M. (2018). Health of transgender adults in the US, 2014–2016. American journal of preventive medicine, 55(3), 336-344.  Lee, H., Operario, D., van den Berg, J. J., Yi, H., Choo, S., & Kim, S. S. (2020). Health disparities among transgender adults in South Korea. Asia Pacific Journal of Public Health, 32(2-3), 103-110.  Thoma, B. C., Salk, R. H., Choukas-Bradley, S., Goldstein, T. R., Levine, M. D., & Marshal, M. P. (2019). Suicidality disparities between transgender and cisgender adolescents. Pediatrics, 144(5). | We thank the reviewer for the comment. For clarification, we used cisgender males as the reference group simply as a matter of convention (as per Johns et al., 2019)  Johns MM, Lowry R, Andrzejewski J, Barrios LC, Demissie Z, McManus T, et al. Transgender Identity and Experiences of Violence Victimization, Substance Use, Suicide Risk, and Sexual Risk Behaviors Among High School Students - 19 States and Large Urban School Districts, 2017. MMWR Morb Mortal Wkly Rep. 2019 Jan 25;68(3):67–71.  However, upon reviewing the reviewer’s suggested reference, we agree that it would be better to revise the analyses and also include measure of association with cisgender girls as an additional reference group. Tables 3 and 4 revised.  We also added the following remarks to the Data Analysis sub-section of METHODS:  “In a similar manner to previous studies on health disparities among transgender people [19–21], we compared the prevalence of each outcome in each transgender group to the prevalence among cisgender boys and cisgender girls separately.”  Changes made to Table 3 and (newly created) Table 4 |
| Comment 5A.  The paragraph that starts at line 368 in page 30 should be revised. The paragraph is not well-organized and contains statements that are concerning. First, the author makes unnecessary comparisons with other countries (e.g. US). Since the study is not about making international comparisons of transgender youth health status, these comparisons seem unnecessary. Furthermore, the comparison sentence colludes with the findings. Table 3 shows that transgender female and transgender male adolescents have 1.82 times and 2.06 times higher odds of attempting suicide compared to cisgender male adolescents. This result, along with results of other indicators of mental health, suggests that the mental health disparities between cisgender and transgender adolescents in Thailand are severe. Despite the severity of health disparities, the author writes that suicidality in transgender adolescents is lower in Thailand when compared to other countries only to highlight the culture of acceptance of transgender identity in Thailand. The reviewer is concerned that these sentences that make comparisons may downplay the apparent severity of the mental health gap experienced by transgender adolescents in Thailand. | We thank the reviewer for the comments.  We have removed comparisons with other countries. We have also removed remarks that collude with the findings.  We agree that the sentences that we wrote in the first draft can downplay the severity of mental health gap and have revised the paragraph accordingly. |
| Comment 5B.  In the same paragraph from the comment above, the author seeks to explain the differences in mental health outcomes between transgender male and transgender female adolescents without evidence and proper citation. If authors want to discuss differences between the two populations, authors should carry out statistical analyses that directly compares the health of transgender male and transgender female adolescents to assess whether there are statistically significant differences between the two groups. | We thank the reviewer for the comments. We decided to remove remarks regarding differences in mental health outcomes between transgender boys and transgender girls, as our analyses focus on the disparity between transgender and cisgender adolescents and these discussions without evidence and proper citation digress the discussion from the focus. |
| ***Minor comments |  |
| Comment 1.  Table 2 shows the distribution of basic characteristics among study respondents by their gender identities. In addition to representing the distribution of the characteristics, I suggest that the author includes results of chi-square tests, to show any significantly different distributions of these characteristics among participants of different gender identities. | We have added p-values from Chi-square tests with Rao-Scott adjustments |
| Comment 2.  Past research on health disparities between cisgender and transgender individuals have included measures of income or social status in their lists of covariates (Downing & Przedworski, 2018; Lee et al., 2020; Thoma et al., 2019). It is suggested that the author includes an indicator of socioeconomic status such as household income (Downing & Przedworski, 2018; Lee et al., 2020) or perceived social status (Thoma et al., 2019) as another covariate.  Downing, J. M., & Przedworski, J. M. (2018). Health of transgender adults in the US, 2014–2016. American journal of preventive medicine, 55(3), 336-344.  Lee, H., Operario, D., van den Berg, J. J., Yi, H., Choo, S., & Kim, S. S. (2020). Health disparities among transgender adults in South Korea. Asia Pacific Journal of Public Health, 32(2-3), 103-110.  Thoma, B. C., Salk, R. H., Choukas-Bradley, S., Goldstein, T. R., Levine, M. D., & Marshal, M. P. (2019). Suicidality disparities between transgender and cisgender adolescents. Pediatrics, 144(5). | We thank the reviewer for the comment. Unfortunately, we did not have direct measurement of household income in the data set. We have already adjusted for living situation, which can serve as a crude indicator of household socioeconomic status. However, question A13 asked about the respondent’s monthly stipend (“How much money do you receive as expenses each month?”). The differences between groups with regard to monthly stipend were statistically significant, thus we have included monthly stipend as a covariable in multivariate modified Poisson regression analyses. |
| Comment 3.  Depending on the outcome variable, there were varying numbers of participants dropped due to missing values. This leads to each regression having different sample sizes. Please include sample sizes, frequencies, and the proportions (%) in each of the relevant cells in tables 1, 2, and 3 to clearly provide the results with missing data. | Sample sizes included in Tables 1 and 3. Missing data regarding demographics were presented as “unknown” in Table 2 and thus the remark is not applicable for Table 2.  When we were checking the codes, we realized that we treated missing values as “No” in the previous version of the manuscript (unlike what we described in the Methods section). We have revised Tables 3 and 4 extensively. |
| Comment 4.  In the following sentence, “We then performed multivariate analyses to measure the association between gender classification and the outcomes adjusting for the effect of basic characteristics of the respondents using multivariate logistic regression (lines 244-246, page 14),” please include a phrase or sentence stating that the multivariate analyses were performed separately for each of the outcome variables. | We have revised the sentence to the following:  “We then performed multivariate analyses to measure the association between gender classification and the outcomes adjusting for the effect of basic characteristics of the respondents using modified Poisson regression. The analyses were performed separately for each of the outcome variables.” |
| Comment 5.  Please check the consistency of variable names the author used throughout the manuscript. For instance, the author used the word ‘suicidal ideation’ in the abstract and in other parts, ‘suicide contemplation’ in the method part, and ‘considered suicide’ in table 3. And in checking for the consistency, please refer to prior research for most commonly used terminology regarding your outcomes. For example, regarding suicidal behaviors, the most commonly used terminologies include ‘suicidal ideation’ and ‘suicide attempt.’ | The terms have been changed to “suicidal ideation”, “suicide planning” and “suicide attempt” throughout the manuscript. We retained the term “suicidality” to refer to all 3 outcomes (ideation, planning, attempt) as a group. |
| Comment 6.  There are various health outcomes and health behaviors that have high prevalence, such as 51.1% (ever drinker among transgender male adolescents) and 22.6% (prevalence of depressive experiences among transgender male adolescents). However, the author has used logistic regression for all of their analyses and reports adjusted odds ratios, which would lead to overestimation of the associations. It is suggested that the author runs a modified Poisson regression instead of logistic regression, according to the work of Zou (2004).  Zou, G. (2004). A modified Poisson regression approach to prospective studies with binary data. American Journal of Epidemiology, 159(7), 702-706. | We agree with the reviewer’s comments and the content in Zou’s article, and decided to change the models from multivariate logistic regression models (to calculate Adjusted ORs and 95% CI) to multivariate modified Poisson regression models. However, in consideration of the cross-sectional study design, and based on interpretation of log-binomial regression models output in cross-sectional studies, we referred to the outputs in our study as “Adjusted Prevalence Ratios (PRs) |
| Comment 7.  In Table 2 & 3, The N’s in the second row total out to 38,186, which is different from the n given in the title (38,189). Please check the numbers. | We have revised our analyses. Tables changed extensively. |
| Comment 8.  In United States Center for Disease Control and Prevention, the overall definition of Intimate partner violence is “physical violence, sexual violence, stalking and psychological aggression (including coercive tactics) by current or former intimate partner (i.e., spouse, boyfriend/girlfriend, dating partner, or ongoing sexual partner).” (Breiding, 2015, p. 11)  The perpetrators of IPV defined in your study are “significant other”, which may include family, relative, friend, or acquaintance as well as partner or spouse so on. Therefore, It is recommended to change the term to “Interpersonal violence” rather than IPV, which is used as an idiom.  Breiding, M., Basile, K. C., Smith, S. G., Black, M. C., & Mahendra, R. R. (2015). Intimate partner violence surveillance: Uniform definitions and recommended data elements. Version 2.0. | We thank the reviewer for the comment. We checked the questionnaire and the original word in Thai was *faen*, which refers to boyfriend/girlfriend/intimate partner. Thus we believe that maintaining the term “Intimate Partner Violence” would be appropriate. All mentions of “significant others” in the manuscript were translations of the word *faen*. Changes made from “significant other” to “intimate partner” throughout the manuscript. We apologize for any inconvenience that the mistranslation may have caused. |
| Comment 9.  Please match intext citation styles. | We have checked the manuscript and made changes accordingly. |
| Comment 10.  The study shows that Thai transgender adolescents experience severe disparities in health compared to their cisgender counterparts. Please provide policy implications and/or specific programs to address this public health issue. It is also helpful to provide examples of policies and programs. | Mentions of specific programs to address disparities in behavioral health and experience of violence added to the DISCUSSION section. |
| Comment 11.  There is no discussion on the disparities in experiences of violence between cisgender and transgender adolescents in Thailand. Please provide discussions according to the findings. | We have added a paragraph with discussion on disparities in experiences of violence between transgender and cisgender youths. |
| Comment 12.  The reasons for including ever having had sexual experience and having 4 or more partners as indicators of health-related behavior are unclear. Please provide necessary rationales for including these outcomes or consider dropping them from the analysis. | We believe that it is important to assess the prevalence of being sexually active when studying adolescent health. We intended to use having 4 or more partners as proxy indicator for high-risk sexual practice. However, we understand that number of partners does not necessarily correlates with risky sexual practices and thus we decided to drop having 4 or more partners from the analysis. |
| Comment 13.  Given that “male’’ and “female” are adjectives, please denote the noun that ‘male’ and ‘female’ are explaining (e.g. transgender males is wrong; it should be transgender male adolescents in this context). | Wording changed to “cisgender/transgender boys” and “cisgender/transgender girls”. Changes made throughout the manuscript. |
| **Reviewer #2** |  |
| This paper covers an important topic, but does it in a clumsy and incomplete manner. It fails to sufficiently engage with the literature about disparities between transgender and cisgender youth that exists in Thailand. The paper's introduction needs to be improved, its methods/procedures section needs to be shortened, the data presented in tables needs to be culled and data that is not used in the results section discussion or overall discussion needs to be removed. The use of 6 categories of youth is confusing and what AMAB and AFAB are is left to the imagination of the reader.  I suggest a very major revision. | The authors would like to take this opportunity to thank the reviewer. We are grateful for the reviewer’s extensive comments, which we have found to be both insightful and helpful in the revision process. We have tried to make changes accordingly.    Another reviewer also made remarks on the use of 6 categories and the confusion regarding those we referred as AMAB and AFAB. We agreed with the other reviewer that we did not have enough information to understand the identity of AMAB and AFAB nor discuss their findings. We have excluded respondents with missing gender information from the analysis, deleted AFAB and AMAB from results tables, and revised the corresponding parts in the METHODS, RESULTS, and DISCUSSION sections. |
| Specific comments: |  |
| Line 39: I think the paragraph should start with an explanation of the word 'gender spectrum', i.e. that gender can be seen as a fluid, multi-dimensional concept, not as a binary term. | The following remark has been included as the first sentence of the INTRODUCTION section:  “Gender spectrum refers to the idea that gender can be seen as a fluid, multidimensional concept, where gender exists anywhere on a continuum that includes male and female [1].” |
| Line 45: sexual behaviors are not necessarily detrimental to the health of adolescents and it looks strange to see them listed on par with drug use and suicidal behaviors. I suggest to add the word 'unsafe' before 'sexual behaviors', to refer to unprotected sexual behaviors that can lead to negative outcomes such as HIV, STI or unwanted pregnancy. | Agree. The word “unsafe” included. Changes made throughout the manuscript. |
| Line 48: there is evidence from Thailand for this statement. See Van Griensven F, Kilmarx PH, Jeeyapant S, Manopaiboon C, Korattana S, Jenkins RA, Uthaivoravit W, Limpakarnjanarat K, Mastro TD. The prevalence of bisexual and homosexual orientation and related health risks among adolescents in northern Thailand. Archives of sexual behavior. 2004 Apr 1;33(2):137-47. | Citation replaced with suggested reference. Revisions made as follow:  “Behavioral health issues affect both transgender and cisgender adolescents, although there are disparities in the prevalence of these behaviors[5]” |
| Line 49-50: there is evidence from Thailand for this statement: Guadamuz TE, Wimonsate W, Varangrat A, Phanuphak P, Jommaroeng R, McNicholl JM, Mock PA, Tappero JW, van Griensven F. HIV prevalence, risk behavior, hormone use and surgical history among transgender persons in Thailand. AIDS and Behavior. 2011 Apr 1;15(3):650-8.  and  Yadegarfard M, Ho R, Bahramabadian F. Influences on loneliness, depression, sexual-risk behaviour and suicidal ideation among Thai transgender youth. Culture, health & sexuality. 2013 Jun 1;15(6):726-37.  and  Boonchooduang N, Louthrenoo O, Likhitweerawong N, Charnsil C, Narkpongphun A. Emotional and behavioral problems among sexual minority youth in Thailand. Asian journal of psychiatry. 2019 Oct 1;45:83-7.  and  Kittiteerasack P, Matthews AK, Steffen A, Corte C, McCreary LL, Bostwick W, Park C, Johnson TP. The influence of minority stress on indicators of suicidality among lesbian, gay, bisexual and transgender adults in Thailand. Journal of Psychiatric and Mental Health Nursing. 2020 Nov 15.  and  Logie CH, Newman PA, Weaver J, Roungkraphon S, Tepjan S. HIV-related stigma and HIV prevention uptake among young men who have sex with men and transgender women in Thailand. AIDS patient care and STDs. 2016 Feb 1;30(2):92-100.  There are a few more papers on transgender and sexual minority youth from Thailand that should be reviewed / mentioned in this section, and I would suggest there is no need to include evidence from transgender people in Canada or USA, as the social and cultural context there is completely different. | We agree with the reviewer. Lines revised with suggested references added as follows:  “Transgender people tend to have higher prevalence of high-risk behavior[6,7], depression[8] and suicidality[8,9].” |
| Line 50-55: See above, it is not true that there is no Thai data on disparities between transgender and cisgender youth. | We agree. Lines removed from the INTRODUCTION section |
| Line 57: Thailand is not uniformly tolerant to transgender people. Please review these papers:  Matzner A. The complexities of acceptance: Thai student attitudes towards kathoey. Crossroads: An Interdisciplinary Journal of Southeast Asian Studies. 2001 Jan 1:71-93.  Ojanen TT, Newman PA, Ratanashevorn R, de Lind van Wijngaarden JW, Tepjan S. Whose paradise? An intersectional perspective on mental health and gender/sexual diversity in Thailand.  de Lind van Wijngaarden JW, Fongkaew K. “Being Born like This, I Have No Right to Make Anybody Listen to Me”: Understanding Different Forms of Stigma among Thai Transgender Women Living with HIV in Thailand. Journal of Homosexuality. 2020 Aug 26:1-8.  Manalastas EJ, Ojanen TT, Torre BA, Ratanashevorn R, Hong BC, Kumaresan V, Veeramuthu V. Homonegativity in southeast Asia: Attitudes toward lesbians and gay men in Indonesia, Malaysia, the Philippines, Singapore, Thailand, and Vietnam. Asia-Pacific Social Science Review. 2017 Jun 1;17(1):25-33. | We would like the thank the reviewer for the kind suggestion. We could not access Matzner’s article or Ojanen’s chapter, but we have included the other references (de Lind van Wijngaarden 2020; Manalastas 2017) to the INTRODUCTION section. The first sentence of the third paragraph of the INTRODUCTION section has been revised as follows:  “Thailand is a middle-income country in Thailand is a middle-income country in South East Asia that still has higher prevalence of homonegativity than other countries in the region[10]. Thai transgender women still report experiences of discrimination and violence[11], despite perception as a safe haven for the LGBTQ community[12].” |
| Line 57-61: I suggest to rewrite this, there are several surveys and studies about Thai Transgender women, but it is true that far less is known about transgender men. | The paragraph has been revised for the most part, but we have included remark about the discrepancies in knowledge about transgender boys vs. transgender girls. |
| Page 6 in the document is empty, suggest to remove. | Change made. |
| Page 8-12: I suggest to remove all this information or make it shorter. There is no need to introduce each and every question asked and explain why these questions were included.  The section on Procedure should be shortened or deleted. | We thank the reviewer for the comment. We shortened the section, but retained what we deemed to be enough information for the analyses to be replicable. Changes made from Instrumentation: Behavioral Health to Procedure. |
| Line 222-230: Important to include a limitation on the methodology used. Important but little known evidence from Thailand has shown very large differences in outcomes depending on which data collection method was used. Self-administered written questionnaires resulted in lower self-reporting of 'shameful' behaviors than when a palm-top / iPad device was used for data collection.  Please see:  Van Griensven F, Naorat S, Kilmarx PH, Jeeyapant S, Manopaiboon C, Chaikummao S, Jenkins RA, Uthaivoravit W, Wasinrapee P, Mock PA, Tappero JW. Palmtop-assisted self-interviewing for the collection of sensitive behavioral data: randomized trial with drug use urine testing. American journal of epidemiology. 2006 Feb 1;163(3):271-8. | The Procedure section has been shortened, and details of data collection inside the classroom have been deleted. The suggested citation’s objective was to assess noninferiority of the use of palmtop compared to audio-computer-assisted self-interviews (and superiority of both methods and self-administered questionnaires over face-to-face interviews). Thus we decided to add remarks to the DISCUSSION section as follows:  “Drug use and unsafe sexual practices are sensitive behaviors whose reports can be influenced by social desirability. A previous study suggested that interviews assisted by electronic devices are non-inferior and potentially superior to the use of self-administered questionnaires in measuring sensitive behaviors[43]. We opted for paper-and-pencil questionnaire in this study because of logistical concerns, but future surveys should consider using tablet-assisted self-interviews or audio-computer-assisted self-interviews as potential alternatives.” |
| Line 262 - beginning of Results:  The AMAB / AFAB categories sound a bit silly, if I may say so. More explanation is needed here. Does it mean these boys and girls are gay/lesbian/bisexual/uncertain about their sexuality? It is useful to explain in the paper somewhere that Thai (at least common Thai) does not have separate words for gender and sexuality, both are referred to as 'phet'. This makes it difficult to interpret/translate. Is it possible to simplify the paper, for example by focusing only on transgender students versus cisgender students? Or is it possible to combine all cisgender students and compare them with all transgender students regardless of gender? Or include AMAB/AFAB as 1 category, transgender men&women as 1 category, and all cisgender youth as 1 category? It feels overwhelming to have so many cells and so many categories, and it becomes hard to see what the focus is and what the result of the analysis suggests. I think more work and discussion needs to go into this analysis, and maybe the focus of the paper should be better grounded in previously conducted research on this issue.  Table 3 is way too long, and contains too much information. It is almost as if the authors included the results of the questionnaire and ask the reader to do their own analysis. I suggest to be more selective in presenting information, and break the table up into several smaller tables, and maybe merge some of the sub-populations. | We thank the reviewer for raising concern on this important issues. Another reviewer also raised a similar concern. As such, we decided to exclude those with missing gender information from the analyses, and deleted the AFAB and AMAB columns from Table 3. We also extensively revised Table 3 and cut parts of Table 3 to create Table 4. Transgender students are now compared with cisgender boys and cisgender girls separately, in line with previous studies. We also revised the relevant parts in the METHODS section.  The author agrees that the word ‘phet’ in Thai can refer to both biological sex and gender. The question for measurement of birth gender was actually “A1.1 *Phet kamnerd*” (Lit. “Birth sex”), whereas the question to measure gender identity was “A1.2 *Phet Withi (Khun Kid Wa Khun Pen Phet…”)* (Lit. “A1.2 Sex according to way of life (you think that your are of the … gender)”). The word “*phet withi*” is not part of vernacular Thai, and thus might not have been readily understood by the participants. Furthermore, in Thai academic circles, the word “*phet withi*” seems to translate to either “sexuality” or “sexual orientation”, and is described as such. Nonetheless, given that the phrase in the bracket suggests measurement of gender identity, the author decided to translate this question into English as “A1.2 Gender Identity (you think that you are…)”. The author has revised the sub-section on exposure measurement as follows (and welcomes the reviewer’s further comment):  “We used the responses of two questions to define the genders in this study (Sub-Section A1: Sex): sex assigned at birth, and gender identity. The first question in Sub-Section A1 measured sex assigned at birth with the question “*A1.1 Sex at Birth*” with two possible answer choices: “1) Male”, and “2) Female”. The second question in Sub-Section A1 measured gender identity with the question “*A1.2 Gender Identity (you think that your gender is...)*” with two possible answer choices: “1) Male”, and “2) Female”.”  The author also added the following remarks to the DISCUSSION section:  “The Thai word in the questionnaire was ‘*phet withi*’, which was not part of vernacular Thai.” |
| Line 308-310: Where does the focus on parental problems come from? Do the authors think that parents are to blame for their child being transgender? Or do they think the transgender identity of the child leads parents to have problems? I do not understand the link. Out the enormous amount of data in Table three, only a few small points are discussed in this section, and this is one of them. I do not think this section is solid enough. | We decided to remove parental problems from the analyses. |
| Line 327-330: This suggests that AMAB may in fact be same-sex attracted youth? Apparently 'party drug' use ('ice') is high among young gay men. There are a few papers on this to review using Thai data. | Remarks pertaining to AMAB and AFAB removed from the DISCUSSION section |
| Line 357: That is nonsense, using 1 study to claim that the percentage of non-normative gender identities in any given population has to be <1%. See the papers about vocational students in Northern Thailand and several other papers from Thailand that have assessed the prevalence of homosexual/bisexual orientation and transgender identities, and they are all much higher than 1%. | We thank the reviewer for the comment. We agree. The remark has been revised as follows:  “Approximately one-sixth of the respondents did not answer the gender identity question. The Thai word in the questionnaire was ‘*phet withi*’, which was not part of vernacular Thai. It is possible that respondents who did not answer the gender identity question either did not understand it or perceived it to be a duplicate of the question pertaining to sex assigned at birth and decided to skip it. Future studies should consider modification of the gender identity measurement question to help reduce this non-response.” |
| Line 367: This is an interesting finding. In comparison, see this paper: https://www.sciencedirect.com/science/ article/abs/pii/S0049089X16308468 | We thank the reviewer for the suggestion. We scanned the suggested reference. Unfortunately, our study was a cross-sectional study and the outcome was GPA at time of survey and not educational attainment at adulthood, as so we decided not to include the suggested reference in the revised manuscript. |
| Line 370-376: When discussing Thai media reporting on LGBTQ people, I suggest to review and reference this paper:  Fongkaew K, Khruataeng A, Unsathit S, Khamphiirathasana M, Jongwisan N, Arlunaek O, Byrne J. “Gay Guys are Shit-Lovers” and “Lesbians are Obsessed With Fingers”: The (Mis) Representation of LGBTIQ People in Thai News Media. Journal of homosexuality. 2019 Jan 28;66(2):260-73. | We thank the reviewer for the comment and suggested reference. We have revised the paragraph accordingly. The suggested reference has been included in the following sentence:  “The Thai media also contains hetero-sexist narratives, harmful and discriminatory rhetoric, and negative portrayals of LGBTIQ people[32].” |
| Line 408-418 should move to the limitations section below. | We further expanded the paragraph to also include remarks regarding measurement methods with suggestion for future studies. As such, we would prefer to keep the paragraph in its current location. |
| Line 413-418: Very good point. | Thank you. |
| Conclusion is very short. If this is what the paper is about, it will be easy to slim it down, removing all other information presented in tables but not discussed. | We have revised the conclusion section. |
| **Reviewer #3** |  |
| Overall, I think that the large sample size, robust sampling strategy, and wide selection of variables makes this study a very worthy contribution to the literature on transgender mental health in Thailand. Below, I make some observations and suggestions that I hope will be helpful to contextualizing the findings better. | The authors would like to thank the reviewer for all the comments. We found them to be very helpful in our revision process and have tried to address them accordingly. |
| Terminology: The terms "transgender male" and "transgender female" are a bit confusing. "Male" and "female" are often used to refer to biological sex rather than self-ascribed gender; having read the abstract, I am still unsure whether "transgender male" and "transgender female" refer to individuals of male or female birth sex, and consider themselves boys or girls. Wonder if the abstract word limit makes it possible to clarify the terms in brief there as well? Also, given that the entire study was on adolescents, it might make sense using "transgender boys," "transgender girls" and "cisgender boys," "transgender girls" respectively (the terms "boys" and "girls" might capture the self-ascribed gender better than the strictly biological terms "male" or "female"). | We agree with the reviewer’s comments and have revised the categories to cisgender boys, cisgender girls, transgender girls, transgender boys |
| Lines (l.) 59 - 67: There are quite a few studies conducted in Thailand especially on violence and depression that you might want to take into account here - they may not use exactly the same kind of disaggregation as the present study, but are nevertheless relevant. In the light of these studies, in particular the statement (l. 59-60) that existing studies are based on small-scale surveys or qualitative data is not exactly accurate (e.g., https://doi.org/10.1080/13691058.2020.1737235 ; https://doi.org/10.1371/journal.pone.0237707 ; https://doi.org/10.1080/10826084.2019.1638936 ; http://unesdoc.unesco.org/images/0022/ 002275/227518e.pdf ; https://so03.tci-thaijo.org/index.php/jpss/article/view/102396) ; Table 5 in the Plan International Thailand report on school bullying that relies on self-reported level of masculinity uses a proxy variable for disaggregating transgender and gender-nonconforming youth from those who are gender conforming. | We thank the reviewer for the comment. Another reviewer has also raised this point and we have revised the INTRODUCTION section accordingly. |
| l. 130 Wonder if the Thai terminology used for "sexual intercourse" made it clear to participants what kind of sex was being asked about? And did the participants understand it in the same way as the researchers? There is some older evidence that many Thai people don't count oral or anal contact as "intercourse" - see p. 33 in the following article: https://doi.org/10.1300/J041v09n02_02 - it might be good to reflect on whether this poses any limitations to the validity of the analyses? I see that later in the article, the Limitations section notes this, but the above article may help to contextualize this concern. | The reviewer raised an important point. The author would like to thank the reviewer for the suggested reference. The sexual activity measurement question was "คุณเคยมีเพศสัมพันธ์หรือไม่". Although the wording was not “ร่วมเพศ” as per the article mentioned by Jackson (1999), we believe that ambiguities in our measurement question could have also posed a problem. We have included the following remark with the citation in the DISCUSSION section:  “A nationwide survey in Thailand showed that half of the respondents considered male-male anal sex as "having sex", and at least one-fourth considered oral-genital contact as "having sex"[42]. Although the mentioned survey used a different reference word for “having sex”, it is possible that such variation in definition of "having sex" also existed among our study participants” |
| l. 142 It may be good to note the limits of a question on contraception in, for example, male-male or female-female sexual acts (is it understood by 1-the survey makers and 2-adolescents to refer to birth control only, or to preventing STDs and HIV as well?). This links to the lowered odds ratio of "trans males" using condoms according to Table 3 - most of them are likely to have had sex with females, so it's (in anatomical terms) female-female sex, where condom use is understandably less common than in male-female or male-male sex). | We thank the reviewer for the comment. The word “birth control” was in the question, but we do not know the extent that the respondents were aware that the question only pertained to contraception and not STD prevention. We have added the following remarks to the DISCUSSION section in the paragraph preceding Strengths and Limitations:  “Furthermore, the question on contraceptive use pertained only to birth control measures. Transgender girls who had sex with members of the same sex (in anatomical terms) might have used condom for STD prevention but not for contraception and thus answered “No”, while others might have understood the question to pertain to both contraception and STD prevention, and might have reported condom use that was actually intended for STD prevention. In that regard, the lower condom use among transgender boys who had sex with members of the same sex (in anatomical terms) was understandable. Future studies should include a question on STD prevention methods in addition to contraception in order to obtain a clearer and more complete measure of sexual behaviors.” |
| Table 1. The high percentage of participants not answering the "gender identity" question might be a result of the participants' confusion in being asked about their identity and yet being given only two response options (because an identity question corresponding to the Thai understanding of self-defined phet might rather give a long list of locally recognized identities, such as man, woman, kathoey, gay, tom, dee, etc. where gender identity and sexual orientation are aspects of the same categorical folk identity construct "phet"- see Table 1 in the Plan International report noted above for an example; my guess would be that those identifying as gay, bi, dee, etc. might be quite prevalent among those not answering the second question on their self-ascribed phet). ... this also provides clues to locally relevant response options to the survey as discussed in lines 338-342. It also calls into question whether the statement in lines 347-349 about homosexual and heterosexual persons of the same gender identity being grouped together is correct, and provides an alternative explanation to the statement in lines 356-360. | We thank the reviewer for the comment. We decided to exclude all participants who did not answer the “gender identity” question from the analyses. Changes made throughout the manuscript. |
| Table 2. Wonder if providing by-row rather than by-column percentages would be more useful to the reader? This would make it easier to compare the prevalence of each gender/sex group across types of demographic groups. | We have thought of this, but as the objective of the study was to assess variations in behavioral health and exposure to violence by gender, we feel that attributes of the participants should also be assessed by gender. We therefore decided to keep the table in column percent format as it appeared. |
| l. 364 The statement of there being little discrimination from teachers is questionable. For example, a World Bank commissioned survey ( http://documents1.worldbank.org/curated/en/ 319291524720667423/pdf/124554-v2-main-report- Economic-Inclusion-of-LGBTI-Groups-in-Thailand- Report-Thai-Version-PUBLIC.pdf ) found that 23% of transgender adults reported having been discriminated in education (p. 40). However, there may be a difference in discrimination from teachers as individuals versus educational institutes as institutions. | We agree with the reviewer. We have included the suggested reference and included a qualitative study as an additional reference. The statement has been revised as follow:  “Transgender female respondents had a higher GPA distribution than other groups except for cisgender females. There has been no empirical study on the underlying reason behind this distinction, but anecdotes suggest that Thai transgender women try to gain acceptance from society by educational and professional achievements[28,29]. The relatively high GPA distribution among transgender females could be a reflection of these endeavors during adolescence. In that regard, the literatures also suggest that Thai transgender people face discrimination and stigma in the educational system[11,30]. Policy makers should continue to encourage academic achievements among Thai transgender youths while ensuring that youths are protected from discrimination and stigma in the educational system.” |
| l. 368-389 The suicidality figures and their possible antecedents should be compared with some of the studies on Thai adolescents I mentioned above. There are also other studies that may be relevant, such as emerging Thai studies on LGBTIQ adults (e.g., https://doi.org/10.1111/jpm.12713 ) or a couple of earlier articles on Thai youth using convenience samples (https://doi.org/10.1080/19361653.2014.910483 ; http://dx.doi.org/10.1080/13691058.2013.784362 ). | We thank the reviewer for the comment and suggested references. We have revised the paragraph on suicidality to focus on literatures pertaining to Thai adolescents. We have also cited the Kittiteerasack et al. (2020) and Yadegarfard et al. (2013; 2013b) as per the reviewer’s suggestions. |
| l. 404 Footnote style citation missing for Katz-Wise et al | We have removed parental behaviors from the analyses. |
