# Supplementary material for: Disparities in behavioral health and experience of violence between cisgender and transgender Thai adolescents
Source: PLoS One. 2021 May 28;16(5):e0252520. doi: 10.1371/journal.pone.0252520 (PMC8162664; doi:10.1371/journal.pone.0252520)
Supplement: Attachment — Submitted filename: Response to Reviewers 20210427.docx [file pone.0252520.s005.docx]

Response to Reviewers

PONE-D-20-21445R1

Disparities in Behavioral Health and Experience of Violence between Cisgender and Transgender Thai Adolescents

| **Comment** | **Response** |
| --- | --- |
| **Journal Requirements:** |  |
| Please review your reference list to ensure that it is complete and correct. If you have cited papers that have been retracted, please include the rationale for doing so in the manuscript text, or remove these references and replace them with relevant current references. Any changes to the reference list should be mentioned in the rebuttal letter that accompanies your revised manuscript. If you need to cite a retracted article, indicate the article’s retracted status in the References list and also include a citation and full reference for the retraction notice.  [Note: HTML markup is below. Please do not edit.] | - |
| **Reviewers' comments:** |  |
| **Reviewer #2:** |  |
| I think you have done a stellar job addressing my (and other reviewers') comments. | Thank you so much |
| I have only two minor points. While I am glad you clarified the issue of acceptance/tolerance of transgender people in Thai society somewhat, I do not think the level of 'homonegativity' in Thailand is higher than in neighboring countries, that would take the statement too far! It is a pity you could not access the Matzner paper I sugggested, it is really fascinating and would provide a theoretical explanation for the contradictions that characterise this issue in Thai society; maybe a colleague with access to paid content can find it for you? | We thank the reviewer for the suggestion. We decided to revise the opening sentence in the third paragraph of the INTRODUCTION section to the following:  “Thailand is a middle-income country in South East Asia that experiences homonegativity, not unlike other countries in the region[10].” |
| Also when describing that half of respondents would consider male-male sex to be 'sex', I would add the word 'only'...  I.e. only half of the participants.... This is an important and striking finding :) | Agree. The sentence has been revised as follows:  “A nationwide survey in Thailand showed that only half of the respondents considered male-male anal sex as "having sex", while one-fourth considered oral-genital contact as "having sex"[42]” |
| I like it that you have include linguistic challenges regarding gender/sexuality and agree that sexual orientation is another complicating factor. | Thank you |
| In general, I suggest a final edit by a native English speaker, and then I think the paper should be ready for publication.  Best wishes  Jan ยันต์ | Thank you very much for the compliments and constructive comments ขอบคุณครับ |
| **Reviewer #3** |  |
| I feel the article is overall in much better shape now, following the authors' extensive edits to the manuscript. My comments below are mostly minor language edits and corrections to wordings the authors may have forgotten to adjust following overall changes to the manuscript. Once these have been fixed, I think the paper will be ready for publication. | Thank you. The other reviewer, a native speaker of English, has graciously provided detailed instructions for language edits, which we have followed and made changes throughout the manuscript. |
| **Minor comments:** |  |
| **Abstract:** |  |
| - "identified as transgenders" -> "identified as transgender"  - "higher prevalence suicidal ideation" -> "had a higher prevalence of suicidal ideation"  - "school-going youths population" -> "the school-going youth population" | Thank you for the suggestions. We have made changes accordingly. |
| **Introduction:** |  |
| - watch out for unnecessary use of "still" (e.g., l. 58, 59) | The first two sentences of the third paragraph of the INTRODUCTION section has been revised as follows:  “Thailand is a middle-income country in South East Asia that experiences homonegativity, not unlike other countries in the region[10]. Although many perceive the country as a safe haven[11], Thai transgender women do report experiences of discrimination and violence[12].” |
| l. 56: Would be helpful to clarify what kinds of risk behavior. Sexual? Substances? Extreme sports? | To avoid redundancy in mentioning citation [5] twice, we changed the middle part of the second paragraph to the following:  “Behavioral health issues affect both transgender and cisgender adolescents, although a previous study has shown disparities in the prevalence of unsafe sexual practices, sexual violence, drug use, and depression [5].” |
| l. 58-59: "still has higher prevalence of homonegativity..." To represent Manalastas et al. more accurately, you could state that the level of homonegative attitudes in Thailand is higher than in some other countries in the region, and lower than in others (better still, state which SE Asian countries included in the study are more and which countries are less homonegative than Thailand) | We thank the reviewer for the comment. The other reviewer has also commented on this sentence and remarked that they “do not think the level of 'homonegativity' in Thailand is higher than in neighboring countries, that would take the statement too far!...”  Therefore, as a compromise, we decided to revise the first two sentences of the third paragraph of the INTRODUCTION section to the following:  “Thailand is a middle-income country in South East Asia that experiences homonegativity, not unlike other countries in the region[10]. Although many perceive the country as a safe haven[11], Thai transgender women do report experiences of discrimination and violence[12].” |
| l. 60 "despite perception as a safe haven" -> "although many perceive it as a safe haven" | Change made |
| l 61 "transgender youths are" -> "transgender youth" | Sentence revised as follows:  “Although many perceive the country as a safe haven[11], Thai transgender women do report experiences of discrimination and violence[12].” |
| l. 68-69 "as well as parental issues related to addiction and violence," -> remove if no longer included in analyses | Removal made |
| l. 72 "sex assigned at birth (sex)" -> "sex assigned at birth" | Change made |
| l. 74 "and parental problematic behaviors" -> remove if no longer included in analyses | Removal made |
| l. 116 Does "withdrawal" here refer to the withdrawal method of contraception (coitus interruptus)? Please clarify if the original survey item implied this. | Yes. The most direct translation of the question item would be “external ejaculation” (“หลั่งภายนอก”), which (I believe) corresponds to *coitus interruptus*. We have revised the term to the following:  “Withdrawal (*coitus interruptus*)” |
| l. 141 "Sexual violence" -> "sexual violence" | Change made |
| l. 167-168 "and parental addictive and violent behaviors)" -> remove if no longer included | Change made |
| l. 196-197 "Transgender females and transgender males" -> adjust in keeping with revised terminology | Changed to “Transgender girls and transgender boys” |
| l. 223 "had significantly prevalence of lifetime" -> "had significantly higher prevalence of lifetime" | Change made |
| l. 259 "What gender do you identify as?" - in this case, which Thai words would you use to translate "gender" and "identify" so as to understandable to school-age kids? If you have a suggestion for these, you might include the transcription of your preferred Thai words for these terms in brackets after the word; this would be useful to other researchers | That’s an interesting question. We feel that the word "ตัวตน" would be an appropriate translation of "identity" for the target population. Actually, we changed the wording in the 2020 student health survey questionnaire from "เพศวิถี (คุณคิดว่าคุณเป็นเพศ...)" to "เพศวิถี (ตัวตนคุณที่แท้จริงเป็นเพศใด)". During the pilot-testing of the questionnaire, no student expressed confusion with regard to the question wording. That said, we feel that such suggestion using the word ตัวตน alone without its context would not be as useful to future researchers. Thus we have included the transcription of the original and preferred Thai words in their sentences in the revised manuscript as follows:  “Future studies should consider modification of the gender identity measurement question to help reduce this non-response, e.g., changing from "You think you are..." ("*Khun kid waa khun ben phet*...") to "What gender do you identify as?" ("*Tuaton tii tae jing khun ben phet dai*") to reflect the notion that gender identity is firmly felt and integral to one's being” |
| l. 263 Considering current developments of gender identity in Thailand, including "non-binary" as a response option would also make sense | Change made |
| l. 290 "minority stress" (you could give a brief definition for it) | Sentenced revised as follows:  “A previous study found minority stress (i.e., stress faced by members of stigmatized minority groups caused by factors such as lack of social support, low socioeconomic status, interpersonal prejudice, and discrimination) in the Thai homosexual and bisexual men population[33].” |
| l. 294-295 change terminology to match the rest of the article (transgender males->transgender boys; cisgender males->cisgender boys; transgender females->transgender girls) | Changes made |
| l. 297 "transgender students" -> "transgender boys" (their aspiration to masculinity is the key point here) | Change made |
| l. 316 "These higher" -> "The higher" | Change made |
| l. 322 "the 4P Project" - instead of the cited popular-press article, please cite the project report, which has now been completed and is available at https://lsed.tu.ac.th/uploads/lsed/pdf/research/%20LGBT4P.pdf | We have added the new reference to replace the existing one |
